# *Enterococcus faecalis* Adapts to Antimicrobial Conjugated Oligoelectrolytes by Lipid Rearrangement and Differential Expression of Membrane Stress Response Genes

**DOI:** 10.3389/fmicb.2020.00155

**Published:** 2020-02-14

**Authors:** Gayatri Shankar Chilambi, Jamie Hinks, Artur Matysik, Xinyi Zhu, Pei Yi Choo, Xianghui Liu, Mary B. Chan-Park, Guillermo C. Bazan, Kimberly A. Kline, Scott A. Rice

**Affiliations:** ^1^Interdisciplinary Graduate School, Nanyang Technological University, Singapore, Singapore; ^2^Singapore Centre for Environmental Life Sciences Engineering, Nanyang Technological University, Singapore, Singapore; ^3^School of Chemical and Biomedical Engineering, College of Engineering, Nanyang Technological University, Singapore, Singapore; ^4^Center for Polymers and Organic Solids, Department of Chemistry and Biochemistry and Materials, University of California, Santa Barbara, Santa Barbara, CA, United States; ^5^School of Biological Sciences, Nanyang Technological University, Singapore, Singapore; ^6^The ithree Institute, University of Technology Sydney, Sydney, NSW, Australia

**Keywords:** *Enterococcus faecalis*, conjugated oligolectrolytes, daptomycin resistance, *liaFSR*, cell membrane stress response

## Abstract

Conjugated oligoelectrolytes (COEs) are emerging antimicrobials with broad spectrum activity against Gram positive and Gram negative bacteria as well as fungi. Our previous *in vitro* evolution studies using *Enterococcus faecalis* grown in the presence of two related COEs (COE1-3C and COE1-3Py) led to the emergence of mutants (changes in *liaF* and *liaR*) with a moderate 4- to16-fold increased resistance to COEs. The contribution of *liaF* and *liaR* mutations to COE resistance was confirmed by complementation of the mutants, which restored sensitivity to COEs. To better understand the cellular target of COEs, and the mechanism of resistance to COEs, transcriptional changes associated with resistance in the evolved mutants were investigated in this study. The differentially transcribed genes encoded membrane transporters, in addition to proteins associated with cell envelope synthesis and stress responses. Genes encoding membrane transport proteins from the ATP binding cassette superfamily were the most significantly induced or repressed in COE tolerant mutants compared to the wild type when exposed to COEs. Additionally, differences in the membrane localization of a lipophilic dye in *E. faecalis* exposed to COEs suggested that resistance was associated with lipid rearrangement in the cell membrane. The membrane adaptation to COEs in EFC3C and EFC3Py resulted in an improved tolerance to bile salt and sodium chloride stress. Overall, this study showed that bacterial cell membranes are the primary target of COEs and that *E. faecalis* adapts to membrane interacting COE molecules by both lipid rearrangement and changes in membrane transporter activity. The level of resistance to COEs suggests that *E. faecalis* does not have a specific response pathway to elicit resistance against these molecules and this is supported by the rather broad and diverse suite of genes that are induced upon COE exposure as well as cross-resistance to membrane perturbing stressors.

## Introduction

Enterococci are commensal bacteria commonly found in the gut microflora of humans and animals (Van Tyne and Gilmore, 2014). The two most clinically relevant enterococcal species, *Enterococcus faecalis* and *Enterococcus faecium* (Facklam et al., 2002), are capable of forming biofilms on both biotic and abiotic surfaces (Hashem et al., 2017). Enterococci are primarily associated with hospital associated infections (HAIs) such as opportunistic, catheter-associated urinary tract infections (CAUTIs), other medical device associated biofilm infections and surgical site infections (SSIs) (Tien et al., 2017). Enterococci have malleable genomes and the ability to acquire and transfer antibiotic resistance, which makes infections by *E. faecalis* difficult to treat (Miller et al., 2014). A number of emerging antimicrobial agents in various stages of development are active against Gram positive pathogens including Enterococci. Many of these emerging antimicrobial drugs target cell envelope constituents including lipid II, sortases, lipoteichoic acid, and teichoic acid, among others (van Harten et al., 2017). While many of those compounds are promising as new therapeutics, the development of resistance remains as an issue for both the application of the compounds and for the companies who invest in their development. For example, high levels of daptomycin (DAP) resistance have been observed, which is particularly concerning as DAP was typically used only as a drug of last resort for enterococcal infections (Diaz et al., 2014; van Harten et al., 2017). Accordingly, there is an urgent need to continue to develop new drugs that are active against multidrug resistant (MDR) pathogens but which also elicit minimal resistance in target pathogens.

Conjugated oligoelectrolytes (COEs) (shown in Table 1) are characterized by a π-conjugated aromatic backbone with terminal ionic pendant groups (Thomas et al., 2013). They are amphipathic and are active against a range of pathogens, which includes both gram types, fungi and drug resistant strains (Chilambi et al., 2018). COEs are primarily thought to be membrane acting because they spontaneously intercalate with, and appear to reside in, the lipid bilayer because of their amphiphilic nature (Hinks et al., 2015). Conceptually, COEs share similarities to antimicrobial peptides although their interaction with microbial membranes circumvent some charge based modifications to the cellular envelope that impart resistance to some AMPs in *E. facealis* (Yan et al., 2016). Membrane destruction is apparent in cells treated with antimicrobial COEs and structural defects such as pore formation (Hinks et al., 2015), polar depressions, cell ruptures (Hinks et al., 2014), and membrane fissures (Zhou et al., 2018) are evident along with the leakage of intracellular enzymes (Hinks et al., 2014). There is some evidence of lipid specificity between COEs in membrane perturbation which is reflected in the apparent polar disruption of some COEs compared with the longitudinal fissures caused by others and by the difference in optical properties of COEs in different lipid mixtures observed (Hinks et al., 2015; Bhardwaj et al., 2016). COE1-3C [1,4-bis(4″-(*N*,*N*-bis(6″″-(*N*,*N*,*N*-trimethylammonium)hexyl)amino)-styryl)benzene tetraiodide] and COE1-3Py [1,4-bis(4″-(*N*,*N*-bis(6″″-(pyridinium)hexyl)amino)-styryl)benzene tetraiodide] inhibit *E. faecalis* OG1RF at 2 μM and 1 μM, respectively. In a previous study, serial passaging of *E. faecalis* with either COE produced strains EFC3C and EFC3Py with an increase of 4- and 16-fold in their resistance to COE1-3C and COE1-3Py, respectively, in comparison to the parental strain (Chilambi et al., 2018). Whole genome sequencing of EFC3C and EFC3Py revealed mutations in the *liaFSR* three-component signal transduction system (Chilambi et al., 2018). Quantification of the membrane fatty acid composition of the resistant strains showed significant changes in the *cis*-vaccenic acid and cyclopropane fatty acids in their membranes (Chilambi et al., 2018). These fatty acids were directly implicated in COE resistance in *E. faecalis* through a supplementation study (Chilambi et al., 2018) but they did not explain COE resistance in its entirety. To identify additional factors, which could contribute to this resistance, transcriptomic studies on the response of *E. faecalis* wild type, EFC3C and EFC3Py to COEs were undertaken.

**TABLE 1:** List of bacterial strains used in this study and their characteristics.

**Strain**	**Genotypic characteristics**	**References**
*Enterococcus faecalis* OG1RF	Wild type	Chilambi et al., 2018
*E. faecalis* EFC3C	COE1-3C resistant *E. faecalis* [in frame deletion in *liaF* at position 179 and a substitution in the intergenic region between *treB* (PTS family trehalose porter, IIBC component) and *gloA6* (lactoylglutathione lyase)]	Chilambi et al., 2018
*E. faecalis* EFC3Py	COE1-3Py resistant *E. faecalis* (a non-synonymous substitution at position 98 in the *liaR* region of EFC3Py and mutations in *merR*, a transcriptional regulator, site specific tyrosine recombinase *xerD*, and ATP-binding protein region of a multidrug ABC transporter)	Chilambi et al., 2018
EFC3Py: p*liaR*	EFC3Py derivative complemented with pAL1 carrying wild type *liaR_*OG*__1__*RF*_*	This study
*E. faecalis* OG1RF *liaF_Δ *Ile*__179_*	*E. faecalis* OG1RF derivative with an in-frame deletion in isoleucine at position 179	This study
*E. faecalis* OG1RF *liaF_Δ *Ile*__179_:*p*liaF*	*E. faecalis* OG1RF *liaF_Δ *Ile*__179_* derivative complemented with pGCP123 carrying wild type *liaF*	This study

## Results and Discussion

### Influence of *liaF* and *liaR* Mutations in EFC3C and EFC3Py, Respectively, on Sensitivity to COEs

The genomes of COE resistant *E. faecalis* strains EFC3C and EFC3Py were previously sequenced, revealing mutations in *liaF* (Δ*Ile179*) and *liaR* (A98V), respectively (Table 1) (Chilambi et al., 2018). In addition, strain EFC3C has mutations in the intergenic region between *treB* and *gloA6*, while the EFC3Py strain contained additional mutations in *merR* and *xerD* (Table 1) (Chilambi et al., 2018). LiaFSR is a three-component system that is conserved in Gram positive bacteria and functions as a damage sensing and signal transducing system (Munita et al., 2012). To determine if mutations in these genes confer resistance to COEs, isogenic mutants were made and complemented with the functional genes in this study. Specifically, in this study an isogenic *liaF_Δ *Ile*__179_* mutation in *E. faecalis* OG1RF was generated and complemented with the wild type on a plasmid. Although deletion of *E. faecalis* OG1RF *liaR* was previously reported (Reyes et al., 2015), we were unable to generate an *E. faecalis* OG1RF *liaR*_A__98__V_ mutant, identical to that which arose in EFC3Py. Therefore, to study the influence of *liaR*_A__98__V_ on resistance to COEs, EFC3Py was instead complemented with a wild type allele of *liaR.* MIC determination (Table 2) confirmed that deletion of the isoleucine residue at position 179 of *liaF* was associated with COE1-3C susceptibility. Furthermore, complementation of *E. faecalis* OG1RF *liaF_Δ *Ile*__179_* with the wild type *liaF* restored wild type sensitivity to COE1-3C. Complementation of EFC3Py with *liaR_*OG*__1__*RF*_* showed a twofold increase in MIC against COE1-3C. Similarly, expression of *liaR_*OG*__1__*RF*_* in EFC3Py restored sensitivity to EFC3Py (Table 2). In contrast, complementation of EFC3Py with *liaR_*OG*__1__*RF*_* did not restore sensitivity to COE1-3C (Table 2). EFC3Py contains three background SNPs, none of which contributed to resistance to COE1-3C when present as single mutations (Chilambi et al., 2018). We therefore speculate that, while no single one of those mutants confer resistance to COE1-3C in the *liaR* mutant, it is possible that two or three of those mutations in combination do result in resistance, although this remains to be determined. Thus, we have shown here that LiaF is associated with resistance to COE1-3C, while LiaR was associated with resistance to COE1-3Py.

**TABLE 2:** Minimum inhibitory concentrations (MICs) for wild type and evolved strains of *E. faecalis*.

**Strain**	**COE1-3C^*a*^ (μM)**	**COE1 -3Py^*a*^ (μM)**	**DAP (μM)**
*E. faecalis* OG1RF	2	1	1.23
*E. faecalis* OG1RF *xerD_*Ala*__233__*Val*_*	NA	1	1.23
*E. faecalis* OG1RF *xerD_*Ala*__233__*Va*__*l*_ merR_*Val*__134___*Asp*__135__*insValVal*_*	NA	1	1.23
*E. faecalis* EFC3Py^∧∧^	8	16	9.87
*EFC3Py:* p*liaR*	16	1	9.87
*E. faecalis* EFC3C**	8	1	9.87
*E. faecalis* OG1RF *liaF*Δ*Ile179*	4	1	9.87
*E. faecalis* OG1RF *liaFΔIle179:*p*liaF*	2	1	1.23
*E. faecalis* DAP 21^#^	2	1	78.96
*E. faecalis* DAP 22^#^	2	1	78.96

^*a*^Structures of the COEs used here. NA, not applicable. ^∧∧^ and ** isolates collected during the course of the passaging of OG1RF in COE1-3Py or COE1-3C, respectively. ^#^Isolates from Rashid et al. (2017).

The *lia* operon is associated with a resistant phenotype in response to membrane active antimicrobial agents DAP in *E. faecalis* (Tran et al., 2013). Since DAP and COEs appear to share a superficially similar mechanism of action in enterococci (Chilambi et al., 2018), the role of *liaF* and *liaR* mutations in cross resistance to DAP was studied. The MIC of DAP against EFC3C, EFC3Py, EFC3Py:p*liaR*, and OG1RF *liaF_Δ *Ile*__179_* increased eightfold compared to the wild type (Table 2). Complementation of the *liaF_Δ *Ile*__179_* mutant with *liaF* restored DAP sensitivity to wild type levels, suggesting that this effect was mediated by *liaF*. By contrast, complementation of the EFC3Py mutant with *liaR* did not restore the wild type sensitivity to DAP. This was also confirmed by the lack of change in DAP susceptibility of OG1RF mutants with transposon insertions in these genes in comparison to the wild type (Supplementary Table S1). Recent publications also show that DAP resistance in *E. faecalis* may arise independently of LiaFSR by mutations in a two-component system YxdJK, altered function of a putative fatty acid kinase (*dak*) or via incorporation of exogenous fatty acids into the cell membrane (Harp et al., 2016; Miller et al., 2019). Although we did not detect mutations in those genes, we did observe increased levels of *cis*-vaccenic acid in EFC3Py and cyclopropane fatty acids in EFC3C and EFC3Py (Chilambi et al., 2018). Thus, while *liaF_Δ *Ile*__179_* appeared to be directly associated with DAP resistance, *liaR*_A__98__V_ may not be associated with DAP resistance. Overall, the cumulative effect of background mutation to DAP and COE1-3C in our study supports the possibility of a multilayered cell membrane stress response in *E. faecalis* (Miller et al., 2019).

### COEs Induce a Membrane Associated Stress Response in *E. faecalis* OG1RF Wild Type, EFC3C and EFC3Py

**FIGURE 1:**
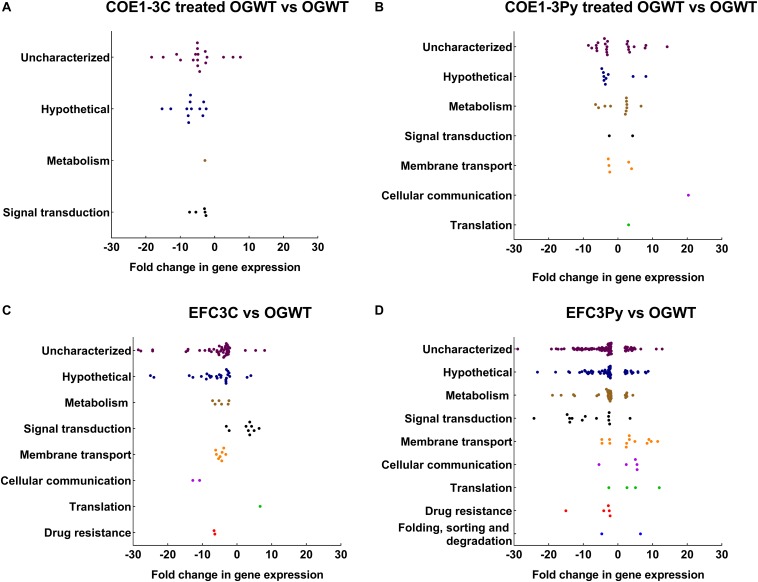
Dot plots representing differentially expressed genes categorized according to the KEGG pathways and their fold change in **(A)** COE1-3C treated, **(B)** COE1-3Py treated *Enterococcus faecalis* wild type (OGWT), **(C)** EFC3C and **(D)** EFC3Py based on KEGG Orthology (KO). The differentially expressed genes which did not fall into any KEGG pathway were assigned as uncharacterized. The fold changes were in comparison to *E. faecalis* OG1RF wild type. False discovery rate (FDR) of 0.05 was chosen for all of the comparisons.

To investigate how mutations in *liaF* and *liaR* mediate resistance to COEs, a transcriptomic approach was undertaken here. The changes in gene expression profiles (induced or repressed ≥ fourfold) of the wild type as well as the COE resistant mutants were compared in the presence or absence of the compounds COE1-3C or COE1-3Py. In both COE1-3C and COE1-3Py treated wild type *E. faecalis* OG1RF, the majority of differentially expressed genes were uncharacterized or hypothetical genes according to KEGG Orthology, apart from those associated with signal transduction (Figure 1). The transcriptomic differences of the untreated mutants, EFC3C and EFC3Py, compared to the wild type, indicated that genes associated with drug resistance, cellular communication, signal transduction, and membrane transport were affected (Figure 1, Supplementary Figures S1, S2 and Supplementary Tables S4–S6).

**FIGURE 2:**
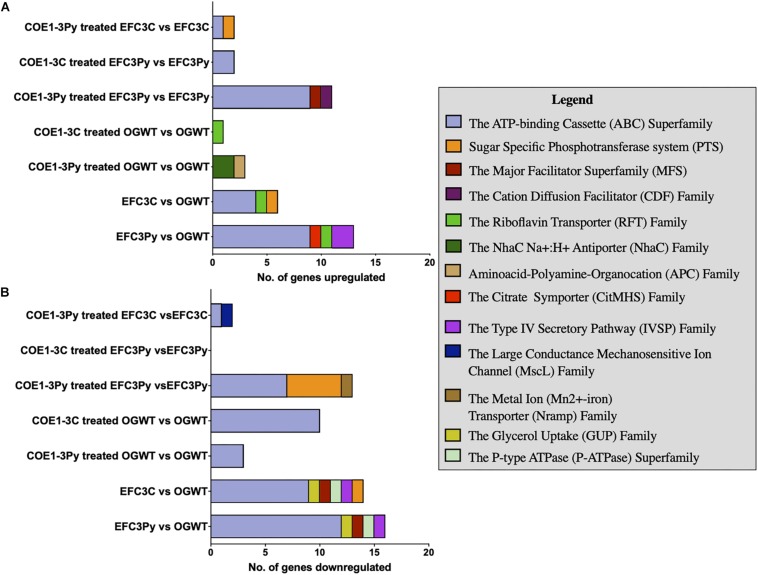
Stacked bar graph plot of the membrane transporter classes **(A)** upregulated and **(B)** downregulated in gene expression in comparison to the *Enterococcus faecalis* OG1RF wild type (OGWT) in relation to COE treatment or in COE resistant mutants. Transporter genes were identified based on analysis using TransporterDB (Elbourne et al., 2017). A false discovery rate (FDR) of 0.05 was used as the threshold. Genes that were altered in expression, ≥ 4 fold and ≤ −4 fold in fold change (FC) were considered in the plot.

Approximately 10% of the ≥ fourfold differentially expressed genes were associated with membrane transport functions. The TransportDB database was used to categorize these according to substrate, family, or membrane transporter class (Elbourne et al., 2017). Genes associated with the ABC class of membrane transporters were differentially expressed in all comparisons (Figure 2). ABC transporters are ubiquitous membrane proteins that couple ATP hydrolysis to the translocation of diverse substrates across cell membranes (Locher et al., 2002). These transporters are typically associated with osmotic stress responses, pathogenesis, lipid transport, and the export of biomolecules (Lubelski et al., 2007).

Induction of a putative glutamate: GABA antiporter (OG1RF_10368, 20.4 fold; OG1RF_11810, 7.9 fold), involved in acid stress response, was observed in wild type cells treated with COE1-3Py in comparison to the untreated strain. The glutamate antiporter increases the cytoplasmic pH by the expulsion of H^+^ ions, with the uptake of glutamate and removal of GABA (Higuchi et al., 1997). The putative glutamate: GABA transporter is associated with response to acid stress conditions in *Escherichia coli*, *Listeria monocytogenes*, and *Lactococcus lactis* (De Biase and Pennacchietti, 2012). Similarly, genes coding for a Na^+^/H^+^ antiporter (OG1RF_10288 and OG1RF_10369), which functions under low pH conditions (Kakinuma, 1987), were upregulated in the presence of COE1-3Py. By contrast, the Resistance/Nodulation/Cell Division (RND) family of secondary transporters (OG1RF_10301 and OG1RF_12269) were downregulated after treatment with COE1-3C in the wild type OG1RF. These secondary transporters use a proton motive force or sodium motive force to export drugs across the membrane (Bolhuis et al., 1997). In the case of COE1-3Py, there is an upregulation of transporters associated with acid stress while in response to COE1-3C, there is a repression of the RND-type efflux pumps and a few ABC transporters. To confirm the role of these membrane transporters on the sensitivity to COEs, MICs of *E. faecalis* mariner transposon library mutants with insertion-deletions at these gene loci was measured. However, the transposon mutants of ABC transporters encoded by OG1RF_12536, OG1RF_10895, OG1RF_10896, OG1RF_10760, and OG1RF_10368 did not show any changes in susceptibility despite being differentially expressed in the transcriptomic analysis. The results seem to show that both COEs compromise the membrane integrity with a varied response to each molecule as seen by the differences in the expression of the membrane transporters.

Genes encoding a multidrug efflux transporter, OG1RF_11766 and OG1RF_11767, were induced 787- and 699-fold, respectively, in EFC3Py. This multidrug efflux transporter was also significantly induced after treatment of EFC3Py with COE1-3C and COE1-3Py. The product of these genes has been associated with the efflux of fluoroquinolone in *E. faecalis* and is involved in efflux of fluorescent substrates such as ethidium bromide and Hoechst 33342 (Hürlimann et al., 2016). The orthologs of OG1RF_11766 and OG1RF_11767 have been shown to be significantly upregulated in *E. faecalis* V583 on treatment with chlorhexidine, a clinically relevant antiseptic (Bhardwaj et al., 2016). It has been shown to be upregulated in Fst toxin-treated *E. faecalis* OG1X, suggesting its association with extracytoplasmic stress response in *E. faecalis* (Brinkman et al., 2013). Similarly, ortholog of EF1057 in *E. faecalis* V583 (OG1RF_10838) was downregulated in response to iron chloride stress while orthologs of EF2226-EF2227 in *E. faecalis* V583 were upregulated in response to chlorohexidine treated *E. faecium* 1,231,410, respectively (López et al., 2012; Bhardwaj et al., 2016). The strong induction of this multidrug efflux transporter in EFC3Py upon treatment with COE 1-3Py, coupled with being the only genes upregulated above the threshold of fourfold change in gene expression in the mutant COE1-3C (Supplementary Tables S7, S8), suggests that OG1RF has upregulated its efflux pump expression in response to the *in vitro* evolution in COE1-3Py. This has been supported by the 14% decrease in the relative uptake of COE1-3Py in EFC3Py than wild type OG1RF (Chilambi et al., 2018). The lipidomic analysis of a deletion mutant of *efrEF*, orthologs of OG1RF_11766 and OG1RF_11767, in *E. faecium* 410 showed that this ABC transporter could be involved in importing lipid species from the media (Bhardwaj et al., 2017). While we previously showed that fatty acid supplementation could partially restore resistance in our COE mutants (Chilambi et al., 2018), it remains to be determined if there are any changes in the membrane lipid compositions in these strains as a result of mutation at OG1RF_11767 which could influence their resistance to DAP and COE1-3C. Thus, the transcriptomic data presented here suggest a general, non-specific stress response of *E. faecalis* upon exposure to the COEs.

### Differential Regulation of Cell Envelope Stress Responses in Untreated EFC3C and EFC3Py

Despite the diversity in the cell envelope stress responses among Gram positive bacteria, two that are commonly associated with membrane associated stress responses in these organisms are the *dlt* operon and penicillin binding proteins (PBPs) (Jordan et al., 2008). The transcriptomic profiles of EFC3C and EFC3Py showed repression of genes involved in D-alanylation of lipoteichoic acid, *dltA-D* (OG1RF_12109–OG1RF_12112) in both EFC3C and EFC3Py (Supplementary Table S4). D-alanylation of lipoteichoic acid is one of the mechanisms by which *E. faecalis* OG1RF resists cationic antimicrobial peptides (CAMPs) (Hinks et al., 2015). The *dltA* mutants of Enterococci are typically sensitive to CAMPs (Fabretti et al., 2006). However, previous studies on COE susceptibility of OG1RXΔ*dltA-D* mutants demonstrated that there was no difference in susceptibility to COE1-3C and COE1-3Py in comparison to the wild type (Hinks et al., 2015; Yan et al., 2016). Therefore, downregulation of the *dlt* operon expression (Supplementary Table S4) in this study could be an indirect effect of the adaptation to COEs and implies that COEs induce a general cell membrane associated stress response in *E. faecalis* and that they interact with cell envelope in a manner distinct from that of CAMPs.

The transcriptomic analysis revealed that PBP 1A (OG1RF_10417), associated with peptidoglycan biosynthesis, was downregulated in both EFC3C and EFC3Py (Supplementary Table S4). In the case of EFC3Py, the cell division protein FtsI or PBP3 (OG1RF_12158) was also repressed (Supplementary Table S6). Overall, this suggests that COE1-3C elicit cell envelope stress via membrane perturbation and COE1-3Py via cell wall interactions.

The genes *liaF*, *liaS*, and *liaR* along with genes controlled by LiaR were downregulated in the mutants EFC3C and EFC3Py. The transcriptomic data revealed that, compared to the wild type *E. faecalis*, *liaF* was downregulated (−5.3 and −9.4-fold, respectively) and *liaS* was downregulated (−4.9 and −10.3-fold, respectively) in EFC3C and EFC3Py, and *liaR* was downregulated in EFC3Py by −9 fold. Three genes, *liaXYZ* (previously known as *yvlD* (putative integral membrane protein), *pspC* (phage shock protein C) and *yvlB* (putative regulator of stress) (Miller et al., 2013) that are putatively regulated by LiaR based *in silico* analysis, were repressed in EFC3C and EFC3Py. In EFC3Py, *yvlD*, *pspC*, and *yvlB* were significantly repressed, −92.2, −102.7, and −215.7, respectively (Supplementary Table S4), while EFC3C showed relatively lower fold changes of −28.5, −32.3, and −64.2, respectively, for the same genes (Supplementary Table S4). The mutations in the EFC3C and EFC3Py mutants lead to differences in the expression levels of *liaFSR*, *yvlB*, *yvlD*, *and pspC* and hence possibly contribute to the observed differences in the MICs of EFC3C and EFC3Py. These data therefore suggest that the two COEs elicit slightly different responses in *E. faecalis*, which is also reflected in the cross-resistance results for the MICs (Table 2) as well as differences in binding membrane lipids for two compounds (Chilambi et al., 2018).

### Membrane Lipid Rearrangement in Response to COEs

Mutations in the *liaFSR* three-component system have been associated with the redistribution of phospholipids in the membrane in response to membrane interacting molecules. Previous studies have shown that COE1-3C and COE1-3Py fluoresce at 566 and 560 nm, respectively (Yan et al., 2015). Since nonyl-acridine orange (NAO), a lipid stain used to visualize cardiolipin (CL or DPG) and phosphatidyl glycerol (PG) domains, has an emission spectrum coinciding with the COEs, FM4-64, a lipophilic membrane stain was used for the purpose of studying lipid rearrangement in our study. The localization of FM4-64 was previously reported to be dispersed in *E. faecalis* strain S613Δ*_*liaF*__177__*gdpD*__170__*cls*__61_*, an *E. faecalis* S613 strain harboring mutations in *liaF* and genes encoding enzymes associated with the phospholipid metabolic pathway (*gdpD* and *cls*). The wild type on the other hand showed focal enrichment of FM4-64 at the septum. The change in localization pattern suggests possible structural changes in membrane architecture and relative changes in phospholipid concentration (a decrease in CL and PG) in S613Δ*_*liaF*__177__*gdpD*__170__*cls*__61_* (Tran et al., 2013).

**FIGURE 3:**
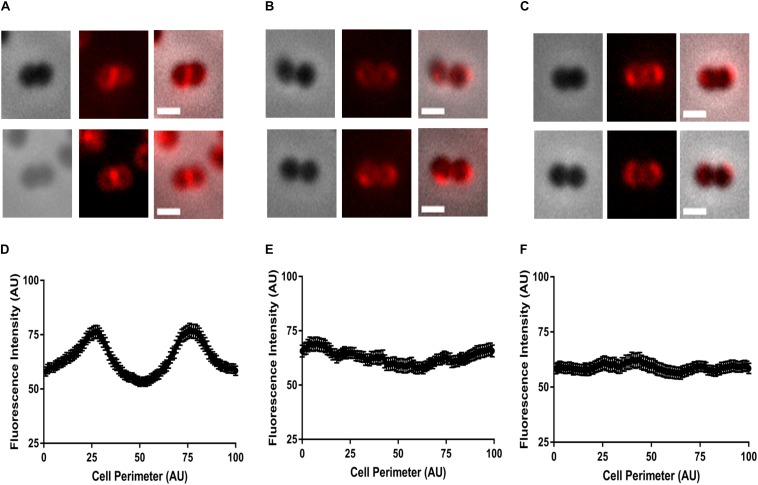
Representative images of localization patterns of FM4-64 in **(A,D)** *Enterococcus faecalis* OG1RF wild type, **(B,E)** EFC3C, and **(C,F)** EFC3Py strains. Fluorescence intensity along the circumference of the cell was plotted against the cell perimeter of 1–100 units to generate a fluorescence distribution profile. Intensity analysis performed by PSICIC analysis software for two independent experiments with duplicates in each experiment. Excitation and emission wavelengths were 515/640 nm, respectively. The scale bar represents 0.5 μm.

Therefore, to determine if COE resistance was associated with such changes in the membrane lipid distribution, we co-stained the cells with FM4-64 and either COE1-3C or COE1-3Py. We quantified the fluorescence intensity profile of FM4-64, COE1-3C, and COE1-3Py labeling along the cell perimeter of individual cells using Projected System of Internal Coordinates from Interpolated Contours (PSICIC) software (Guberman et al., 2008), as described (Kandaswamy et al., 2013). The maximum fluorescence intensity of FM4-64 and both COEs was observed at positions 25 and 75 (Figure 3 and Supplementary Figures S3, S4), which corresponds to either side of the cell septum in the wild type. However, for the mutants EFC3C and EFC3Py, a non-uniform distribution of the fluorescence intensity was observed along the cell periphery, supporting the initial observation that the mutants have undergone membrane lipid rearrangement (Figure 3). Imaging EFC3C and EFC3Py with COE1-3C and COE1-3Py also showed a shift to the cell periphery in comparison to the wild type (Supplementary Figures S3, S4). Previously, it was shown that membrane fatty acid composition changes correlate with increased COE tolerance in *E. faecalis*. The shift in the localization of FM4-64 and COEs toward a non-uniform distribution along the cell periphery suggest that the compositional change in lipid class are also likely to occur in EFC3C and EFC3Py. In addition, these changes are likely to contribute to COE tolerance too, a finding which is supported by the changes in the membrane associated protein expression in the transcriptomic data.

### EFC3C and EFC3Py Are More Resistant to Sodium Chloride and Bile Salts Than *E. faecalis* OG1RF Wild Type

The transcriptomic data suggest COE resistance is achieved through a more general stress response and we observed differences in membrane lipid organization. Therefore, we determined the impact of COE resistance on resistance to two other stressors, bile salts and sodium chloride, that have been previously linked to altered fatty acid composition in enterococci (Solheim et al., 2014; Gaca and Lemos, 2019). For example, we observed upregulation of glycine betaine transporters and downregulation of *mscL* transporters in EFC3C and EFC3Py (Supplementary Tables S7, S8), which have been linked to NaCl stress in *E. faecalis* (Solheim et al., 2014). Here, the dose–response curves show that EFC3C and EFC3Py were more resistant to 0.5–2% bile salts and 0.5–4% sodium chloride than the wild type (Supplementary Figure S5). Overall, this supports our hypothesis, based on the transcriptomic and lipidomic rearrangements, that the mutations in in EFC3C and EFC3Py may represent a general adaptation to membrane stress.

## Conclusion

The molecular topology of COEs has been associated with their ability to either intercalate or disrupt the lipid membranes (Thomas et al., 2015). Both COE1-3C and COE1-3Py function as antimicrobial compounds, with the latter exhibiting a higher degree of membrane perturbation than the former (Chilambi et al., 2018). The serial passaging of *E. faecalis* in increasing concentration of COE1-3C and COE1-3Py led to the emergence of low-level resistance to COEs in EFC3C and EFC3Py via adaptive mutations in the *liaFSR* regulon. These mutations correlate with resistance to COEs, as confirmed by deletion and complementation studies. The results demonstrate that COE1-3C and COE1-3Py induce a subtly different but general membrane stress response, whereby they modulate the cell envelope–COE interactions as well as defense strategies such as ABC transporter induction. However, these factors only help *E. faecalis* to achieve 4–16-fold increase in COEs tolerance, in contrast a 256–512-fold increase is observed in response to *in vitro* adaptation to DAP in *E. faecalis* OG1RF (Table 2). This is a promising feature from a drug discovery view point as it suggests that COEs may have an extended therapeutic lifetime before significant resistance develops. While there seems to be an obvious role of membrane lipids in COE resistance, we have been unable to fully explain this role at a mechanistic and regulatory level. To better understand how the change of an alanine for a valine might impact the function of LiaR, we compared the amino acid sequence of the LiaR from OG1RF with the that of *E. faecium* SD3B-2 (or *E. faecium* R494) since the crystal structure of the entire LiaR sequence of the latter has been published (Davlieva et al., 2016). At the amino acid level, alanine is conserved at position 98 in the two proteins. This mutation is distinct from the more commonly reported mutations in LiaR that are associated with DAP resistance, i.e. W73C. Based on the structure, the A98V substitution observed in EFC3Py is in the receiver binding domain (1-139 aa residues) of LiaR. Both alanine and valine are hydrophobic amino acids and differ only in that valine has an additional –CH_3_ group. It is possible that a bulkier side chain may change the DNA binding properties, folding of the protein or dimerization of the activated protein. Further work that directly tests DNA binding of the wild type OG1RF LiaR and the mutant as well as homology modeling of the OG1RF LiaR with known structures, or direct structural determination may provide insights into how the alanine to valine mutation alters function of LiaR with respect to resistance to molecules such as COEs and DAP. Along these lines, follow-up studies to unify genetic, lipidomic, and proteomic resistance profiles should be a future research direction which could explain COE resistance in more detail. *In vivo*, opportunistic pathogens have been observed to adapt to their host environment by undergoing changes in transcriptional factors without undergoing major genetic mutations (Didelot et al., 2016). This study emphasizes the need to validate the transcriptional response of a pathogen to an antibiotic is essential while studying the antibiotic resistance evolution mechanisms as seen by emergence of improved bile and salt tolerance in EFC3C and EFC3Py despite no direct genetic mutations associated with the same. The differences in gene expression in response to the two COEs is likely to be a reflection of differences in their structures, represented by a terminal pyridinium ion in COE1-3Py, as opposed to the tri-methyl group in COE1-3C and the unique perturbations they induce. This conclusion is supported by our previous study showing that COE1-3Py induced a more complex membrane perturbation and caused the membrane to be more permeable than 3C (Chilambi et al., 2018). Some of the transcriptomic observations, for example, highly upregulated expression of the multidrug efflux transporter (OG1RF_11766–OG1RF_11767) in EFC3Py or downregulation of hyperosmotic stress associated channel, *mscL* in EFC3C corroborate these initial findings. The results indicate that *E. faecalis* responds uniquely to each COE and this is a consequence of minor structural differences between the COEs. This is promising from a medicinal chemistry viewpoint as it points to easily tractable structure activity relationships where simple design modifications can be used to improve the drug-like properties of COEs and eventually in the treatment of a number of infections caused by wild type bacteria, their drug resistant counterparts, and biofilm forming strains.

## Materials and Methods

### Bacterial Strains and Conjugated Oligoelectrolytes

Bacteria (Table 1) were cultured in Brain Heart Infusion (BHI) broth, overnight at 37°C under static conditions. COE1-3C [1,4-bis(4″-(*N*,*N*-bis(6″″-(*N*,*N*,*N*-trimethylammonium)hexyl)- amino)-styryl)benzene tetraiodide] and COE1-3Py [(1,4-bis(4″-(*N*,*N*-bis(6″″-(pyridinium)hexyl)amino)-styryl)benzene tetraiodide)] were synthesized as described previously (Garner et al., 2010; Yan et al., 2016).

### Construction of *liaFΔIle*, *liaFΔIle:pliaF*, *liaR*_A98V_ and *liaR_A98V_:pliaR* Mutants of *E. faecalis* OG1RF

To create in frame deletions of *liaF* in *E. faecalis* OG1RF, 800 bp up- and downstream of the isoleucine at position 179 was amplified from *E. faecalis* EFC3C using the primer pairs *liaF*_INF_F and *liaF*_INF_R (Supplementary Table S9). These primers were used to amplify the *liaF* gene with 15 bp homologous to the ends of the linearized vector and included *Eco*RI and *Hin*dIII restriction endonuclease sites. PCR products were cloned into pGCP213 (Nielsen et al., 2012) and transformed into Stellar^TM^ Competent Cells for selection on LB plates with 750 μg/mL of erythromycin (Erm). Constructs were confirmed by sequencing (Supplementary Table S9). The *E. faecalis* OG1RF *liaF*_Δ *Ile*__179_ mutant was created by transformation of *E. faecalis* OG1RF with the deletion construct pGCP213_*liaF_Δ *Ile*__179_* by electroporation and selection at 30°C on 25 μg/mL of Erm. Double cross-over events, representing deletion mutants, were subsequently identified by passaging at 30°C without Erm and confirmed by sequencing.

The *liaR*_A__98__V_ from EFC3Py was amplified using the primers *liaR*_INF_F and *liaR*_INF_R (Supplementary Table S9). The same procedure as described above was used to derive the pGCP213_ *liaR*_A__98__V_ construct from the Stellar^TM^ Competent Cells. The insertion of *liaR*_A__98__V_ in pGCP213_ *liaR*_A__98__V_ was verified by sequencing and the construct was transformed into *E. faecalis* OG1RF.

For complementation of the mutants, the intact, wild type *liaF* gene, along with its promoter, was amplified from *E. faecalis* OG1RF using the primers (Supplementary Table S9) and strategy as above and cloned into pGCP123. After confirmation by sequencing, the construct was introduced into *E. faecalis* OG1RF *liaF_Δ *Ile*__179_* and the transformants *E. faecalis* OG1RF *liaF_Δ *Ile*__179_*:p*liaF* selected on 500 μg/mL kanamycin plates. The wild type *liaR* gene was cloned using the primers *liaR*_comp_fwd and *liaR*_comp_rev (Supplementary Table S9) and inserted into pAL1, which is derived by inactivating the chloroamphenicol resistance gene in pABG5 (Granok et al., 2000; Kline et al., 2009) for complementation of the EFC3Py mutant. Since the exact location of the native promoter of *liaR* was unknown, it was placed under the control of *rofA*, an inducible promoter (under anaerobic conditions) present in pAL1. After confirmation of the correct insertion, EFC3Py cells were transformed with pAL1_*liaR* and isolated on 500 μg/mL kanamycin plates.

### Minimum Inhibitory Concentration (MIC)

Minimum inhibitory concentration tests with COEs were conducted using broth microdilution based on a method reported earlier (Wiegand et al., 2008; Chilambi et al., 2018), in BHI broth. The MIC of DAP was measured by supplementing BHI medium with 50 μg/mL CaCl_2_. MICs tabulated in Table 2 are the median of multiple experiments (*n* > 3). MIC tests with COEs were conducted using broth microdilution based on a method reported earlier (Wiegand et al., 2008; Chilambi et al., 2018), in BHI broth. The MIC of DAP was measured by supplementing BHI medium with 50 μg/mL CaCl_2__._ MICs tabulated in Table 2 are the median of multiple experiments (*n* > 3).

### Sample Preparation for RNA Extraction

Overnight cultures were diluted 1:100 into 5 mL of BHI medium and grown at 37°C without shaking. The diluted cultures were grown to the mid-log phase and normalized to an optical density (OD_600 *nm*_) of 0.4. Subsequently, COE1-3C or COE1-3Py was added to the normalized cultures at their corresponding MIC as follows: wild type *E. faecalis* OG1RF (2 and 1 μM, respectively), EFC3C (8 and 1 μM, respectively), and EFC3Py (8 and 16 μM, respectively), and incubated for 15 min in triplicate. RNA was extracted from cell pellets centrifuged at 10,000 *g* for 30 s from 1.8 mL of treated culture.

### RNA Extraction

Total RNA was extracted using the moBio Ultraclean microbial extraction kit (MoBio, Carlsbad, CA, United States) according to the manufacturer’s protocol. The isolated RNA was treated with TURBO DNA-free^TM^ kit (Invitrogen Ambion) to remove any traces of DNA. The RNA was purified after DNAse treatment using RNA Clean XP bead suspension (Agencourt Bioscience). The purified RNA was eluted with nuclease-free water (Invitrogen Ambion). Total RNA quantity and purity was determined using a NanoDrop spectrophotometer (Thermo Scientific, DE, United States) and Qubit^®^ RNA HS Assay Kit (Life Technologies) according to the manufacturers’ protocols. The integrity of RNA was measured using a 2200 TapeStation system (Agilent Technologies) with RNA Analysis ScreenTape (Agilent Technologies).

### RNA Sequencing

Library preparation was performed according to the TruSeq Stranded mRNA protocol (Illumina) with the following modifications: The oligo-dT mRNA purification step was omitted and instead, 200 ng of total RNA was directly added to the Elution2-Frag-Prime step. The PCR amplification step, which selectively enriches for library fragments that have adapters ligated on both ends, was performed according to the manufacturer’s recommendation but the number of amplification cycles was reduced to 12. Each library was uniquely dual barcoded with Illumina’s TruSeq HT RNA barcoded adapters to allow pooling of libraries for sequencing. The finished libraries were quantitated using the Picogreen assay (Invitrogen) and the average library size was determined on a Bioanalyzer 2100, using a DNA 7500 chip (Agilent). Library concentrations were then normalized to 4 nM and validated by qPCR on a ViiA-7 real-time thermocycler (Applied Biosystems), using qPCR primers recommended in Illumina’s qPCR protocol, and the PhiX control library as standard (Illumina). The libraries were then pooled at equimolar concentrations and sequenced on an Illumina HiSeq2500 sequencer in rapid mode at a read-length of 100 bp paired-end.

### RNA-Seq Analysis

The total RNA samples were sequenced on Illumina HiSeq2500 sequencer at a read length of 100 bp paired end. Data processing workflow followed slightly modified protocol for differential expression (DE) analysis of RNA sequencing data using R Bioconductor (Anders et al., 2013). Briefly, the quality of Illumina pair-ended reads was first assessed using FastQC (Andrews, 2010). Ribosomal RNA reads were discarded using SortMeRNA (Kopylova et al., 2012) and the remaining non-rRNA sequences were mapped against the OG1RF reference genome (accession number NC_017316.1) using Burrows-Wheeler Alignment software (Li and Durbin, 2009). Aligned data were sorted with SAMTools (Li et al., 2009) and HTSeq was used to extract counts (Anders et al., 2015). RNA reads and extracted counts for each sample were deposited in the NTU open access data repository (DR-NTU^1^). DE analysis was performed using the EdgeR pipeline (Robinson et al., 2010) in R programming language^2^. Counts were normalized to total library size, followed by dispersion estimation and DE test. For DE gene detection, a false discovery rate (FDR) of 0.05 was chosen as the threshold. Fold changes in gene expression of treated isolates were expressed relative to those of the untreated wild type. Gene expression in EFC3C and EFC3Py exposed to either COE1-3C or COE1-3Py was compared to untreated EFC3C and EFC3Py, respectively. DEGs were further enriched based on KEGG pathway analysis (Kanehisa and Goto, 2000). ClusterProfiler R package was used for the analysis and to draw bar and dot plots (Yu et al., 2012).

### Survey of Lipid Rearrangement in EFC3C and EFC3Py With FM4-64 and COEs

Overnight cultures of *E. faecalis* OG1RF, EFC3C, and EFC3Py were normalized to OD_600_ = 0.5 in phosphate buffer saline (PBS, pH 7.4). COE1-3C or COE1-3Py was added to the cultures at the following concentrations: wild type *E. faecalis* OG1RF (1 and 0.5 μM, respectively), EFC3C (1 and 0.5 μM, respectively), and EFC3Py (4 μM of each), incubated for 60 min at 37°C and followed by addition of 10 μg/mL FM^TM^ 4-64FX (ThermoFisher Scientific). After staining, the cells were washed three times in PBS, and 20 μL of cells was spotted onto poly-L-lysine coated slides (Sigma–Aldrich), dried at 46°C in a hybridization oven (ThermoFisher Scientific) for 15 min, mounted with Vectashield mounting medium (Vecalotor Laboratories, Inc.), and imaged using a Zeiss Axio Observer Z1 fitted with a 100×/1.30 oil immersion objective (Carl Zeiss, Göttingen, Germany).

Quantification of fluorescence distribution along the cell was performed using PSICIC (Guberman et al., 2008). Cells with a perimeter > 4.8 μm and ≤ 8 μm were defined as early division cells (Kandaswamy et al., 2013) and chosen for the quantitation of the fluorescence of FM4-64 and COEs. Fluorescence intensity along the circumference of the cell was plotted against the cell perimeter coordinates of 1–100 units to generate a fluorescence distribution profile. Quantitative analysis was performed in two independent experiments.

### Sodium Chloride and Bile Tolerance

Overnight cultures of EFC3C, EFC3Py and *E. faecalis* OG1RF wild type in the stationary phase were diluted to a cell density of 5 × 10^5^ CFU/mL. One hundred μL of this diluted culture was aliquoted into 96-well plates containing 100 μL of BHI serially diluted with different concentrations of bile salts (0, 0.5, 1, 2, 4, and 8%) or sodium chloride (0, 0.5, 1, 2, 4, and 8%). The plates were incubated at 37°C without shaking and optical density at 600 nm (OD_600_) was monitored with an Infinite Pro2000 microplate reader (Tecan) reader. The tolerance of EFC3C, EFC3Py, and *E. faecalis* OG1RF wild type was assessed with the OD_600_ measured at 7 h (mid-log phase). The difference in the effect of the two membrane stressors was represented as a plot of% growth = OD_600_ measured at 7 h/OD_600_ measured at 0 h against the concentrations of bile salts or sodium chloride (NaCl). This experiment was performed in triplicate.

## Data Availability Statement

The datasets generated for this study can be found here: https://doi.org/10.21979/N9/MNHETI.

## Author Contributions

GC, JH, KK, and SR conceived and designed the experiments. GB funded the synthesis of COEs for the project. MC-P provided funding support for RNA sequencing. AM and XL performed the RNA sequencing analysis and wrote the methods section for the same. GC and PC carried out the microscopy experiments. GC performed the MIC tests, RNA extraction, and cloning experiments. XZ contributed to NaCl and bile salts assays. GC wrote the manuscript, with edits by SR, JH, and KK.

## Conflict of Interest

The authors declare that the research was conducted in the absence of any commercial or financial relationships that could be construed as a potential conflict of interest.

## Footnotes

1 https://doi.org/10.21979/N9/MNHETI

2 https://www.R-project.org/
