# Supplementary material for: Enterococcus faecalis Adapts to Antimicrobial Conjugated Oligoelectrolytes by Lipid Rearrangement and Differential Expression of Membrane Stress Response Genes
Source: Front Microbiol. 2020 Feb 14;11:155. doi: 10.3389/fmicb.2020.00155 (PMC7033496; doi:10.3389/fmicb.2020.00155)
Supplement: Supplementary file 1 [file Data_Sheet_1.pdf]

Supplementary Material

Supplementary Table S1. Minimum Inhibitory Concentrations (MIC) for wild type and transposon mutants of genes mutated in EFC3Py.

Gene Locus Tag	Gene Product	Position	Insertion sites	MIC of DAP (µM)
E. faecalis OG1RF	-	-	-	1.23
OG1RF_11765	MerR family transcriptional regulator	1853415	295 nt (Position) /768 nt (Gene length)	1.23
OG1RF_11766	Multidrug ABC superfamily ATP binding cassette transporter, ABC protein	1855237	1414 nt /1728 nt	1.23
OG1RF_11767	ABC superfamily ATP binding cassette transporter, membrane protein	1855713	110 nt/1779 nt	1.23
OG1RF_11462	Membrane protein of hypothetical function, adjacent to PspC	1523233	133 nt/357 nt	1.23

Supplementary Table S2. List of genes differentially expressed in *E. faecalis* OG1RF wild type upon treatment with COE1-3C.

Locus	Description	Fold change
**OG1RF_10301**	RND transporter	-5.0
**OG1RF_10302**	ABC transporter ATP-binding protein	-5.0
**OG1RF_10303**	ABC transporter permease	-5.0
**OG1RF_10878**	adhesin	5.4
**OG1RF_11260**	membrane protein	7.5
**OG1RF_11384**	permease	-5.5
**OG1RF_11385**	peptide ABC transporter ATP-binding protein	-6.2
**OG1RF_11462**	membrane protein	-4.8
**OG1RF_11463**	PspC family transcriptional regulator	-4.9
**OG1RF_12269**	RND transporter	-9.9
**OG1RF_11185**	molybdenum cofactor biosynthesis protein MoaB	-8.5
**OG1RF_10527**	peptide ABC transporter ATP-binding protein	-7.5
**OG1RF_12267**	permease	-6.2
**OG1RF_12268**	ABC transporter ATP-binding protein	-5.8
**Signal Transduction**		
**OG1RF_11656**	peptide ABC transporter permease	-7.3
**OG1RF_11657**	ABC transporter ATP-binding protein	-5.4
**Hypothetical Proteins**		
**OG1RF_10304**	hypothetical protein	-15.3
**OG1RF_10353**	hypothetical protein	-7.0
**OG1RF_10354**	hypothetical protein	-8.1
**OG1RF_10531**	hypothetical protein	-7.6
**OG1RF_10532**	hypothetical protein	-7.1
**OG1RF_11751**	hypothetical protein	-4.3
**OG1RF_10659**	hypothetical protein	-4.2
**OG1RF_11252**	hypothetical protein	-4.7
**OG1RF_11464**	hypothetical protein	-4.0

Supplementary Table S3. List of genes differentially expressed in *E. faecalis* OG1RF wild type on treatment with COE1-3Py.

Locus	Name	Fold change
**OG1RF_10288**	sodium:proton antiporter	4.7
**OG1RF_10367**	decarboxylase	14.2
**OG1RF_10369**	sodium:proton antiporter	8.1
**OG1RF_10526**	permease	-6.1
**OG1RF_11227**	mandelate racemase	-6.3
**OG1RF_11810**	transporter	7.9
**Overview**		
**OG1RF_11314**	katA; catalase	6.7
**Hypothetical protein**		
**OG1RF_10075**	hypothetical protein	-4.0
**OG1RF_12152**	hypothetical protein	4.4
**OG1RF_10659**	hypothetical protein	-4.2
**OG1RF_11252**	hypothetical protein	-4.7
**OG1RF_11464**	hypothetical protein	-4.0
**Nucleotide metabolism**		
**OG1RF_12279**	allantoinase	-6.5
**Cellular communication**		
**OG1RF_10368**	amino acid permease	20.4
**Carbohydrate metabolism**		
**OG1RF_10682**	beta-phosphoglucomutase	-5.6
**Signal transduction**		
**OG1RF_11938**	fumarate reductase flavoprotein subunit	4.2
**OG1RF_11185**	molybdenum cofactor biosynthesis protein MoaB	-8.5
**OG1RF_10527**	peptide ABC transporter ATP-binding protein	-7.5
**OG1RF_12267**	permease	-6.2
**OG1RF_12268**	ABC transporter ATP-binding protein	-5.8

Supplementary Table S4. List of genes differentially expressed that were common to both EFC3C and EFC3Py in comparison to the wild type in the absence of COEs.

**Locus**	**Description**	**Fold change in EFC3Py**	**Fold change in EFC3C**
**OG1RF_12235**	protease	12.8	8.0
**OG1RF_11260**	membrane protein	4.7	5.4
**OG1RF_11377**	conjugal transfer protein TraX	-11.9	-4.4
**OG1RF_10654**	two-component system sensor histidine kinase	-10.4	-4.4
**OG1RF_10707**	DNA repair exonuclease	-12.8	-4.5
**OG1RF_11074**	magnesium-translocating P-type ATPase	-17.3	-4.7
**OG1RF_11072**	transcriptional regulator	-16.3	-5.1
**OG1RF_10328**	peptidase M23	-10.1	-5.2
**OG1RF_11073**	LysR family transcriptional regulator	-15.5	-5.3
**OG1RF_12496**	DNA-binding protein	-12.9	-5.4
**OG1RF_12131**	membrane protein	-10.5	-5.8
**OG1RF_11569**	membrane protein	-11.6	-5.8
**OG1RF_10481**	serine hydrolase	-12.8	-6.7
**OG1RF_12298**	DNA-binding protein	-8.4	-7.4
**OG1RF_11094**	membrane protein	-7.4	-8.0
**OG1RF_12067**	tellurite resistance protein	-19.2	-8.0
**OG1RF_11522**	multidrug MFS transporter	-28.9	-9.2
**OG1RF_12500**	LytR family transcriptional regulator	-33.0	-10.8
**OG1RF_10301**	RND transporter	-11.1	-14.1
**OG1RF_10302**	ABC transporter ATP-binding protein	-11.4	-14.2
**OG1RF_10303**	ABC transporter permease	-11.0	-14.7
**OG1RF_11385**	peptide ABC transporter ATP-binding protein	-79.5	-24.3
**OG1RF_11384**	permease	-67.6	-24.3
**OG1RF_12269**	RND transporter	-94.3	-27.8
**OG1RF_11462**	membrane protein	-92.2	-28.5
**OG1RF_12268**	ABC transporter ATP-binding protein	-115.3	-31.4
**OG1RF_11463**	PspC family transcriptional regulator	-102.7	-32.3
**OG1RF_12267**	permease	-139.9	-39.9
	**Amino acid metabolism**		
**OG1RF_10473**	amidase	-16.4	-6.3
	**Carbohydrate metabolism**		
**OG1RF_11199**	diacetylchitobiose-6-phosphate hydrolase	-12.8	-5.2
	**Glycan biosynthesis and metabolism**		
**OG1RF_10417**	penicillin-binding protein 1A	-15.0	-4.4

	**Lipid metabolism**		
**OG1RF_11521**	alkaline phosphatase	-18.9	-4.4
**OG1RF_11570**	glycerophosphoryl diester phosphodiesterase	-12.5	-7.1
	**Membrane transport**		
**OG1RF_12351**	iron ABC transporter substrate-binding protein	4.9	4.9
	**Metabolism of other amino acids**		
**OG1RF_12112**	D-alanine--poly(phosphoribitol) ligase subunit 1	-13.2	-6.0
**OG1RF_12110**	D-alanine--poly(phosphoribitol) ligase subunit 2	-13.9	-6.4
	**Signal transduction**		
**OG1RF_12212**	two-component system sensor histidine kinase	-10.3	-4.9
**OG1RF_12211**	LuxR family transcriptional regulator	-9.4	-5.3
**OG1RF_12111**	D-alanyl-lipoteichoic acid biosynthesis protein DltB	-13.9	-6.1
**OG1RF_12109**	D-alanyl transfer protein DltD	-14.7	-6.6
**OG1RF_11657**	ABC transporter ATP-binding protein	-24.2	-10.8
**OG1RF_11656**	peptide ABC transporter permease	-33.6	-12.8
	**Translation**		
**OG1RF_10057**	peptide ABC transporter substrate-binding protein	12.0	6.7
	**Hypothetical Protein**		
**OG1RF_10492**	hypothetical protein	8.1	4.0
**OG1RF_11239**	hypothetical protein	-8.9	-4.6
**OG1RF_12328**	hypothetical protein	-8.2	-4.7
**OG1RF_10305**	hypothetical protein	-7.6	-5.7
**OG1RF_11093**	hypothetical protein	-7.2	-7.4
**OG1RF_11031**	hypothetical protein	-14.2	-7.7
**OG1RF_11032**	hypothetical protein	-13.9	-8.0
**OG1RF_10354**	hypothetical protein	-8.6	-8.7
**OG1RF_11751**	hypothetical protein	-18.0	-8.9
**OG1RF_10353**	hypothetical protein	-11.1	-10.3
**OG1RF_11200**	hypothetical protein	-32.9	-12.7
**OG1RF_12066**	hypothetical protein	-38.9	-13.8
**OG1RF_10659**	hypothetical protein	-56.6	-24.0
**OG1RF_10304**	hypothetical protein	-33.0	-25.0
**OG1RF_10532**	hypothetical protein	-71.9	-53.7
**OG1RF_10531**	hypothetical protein	-73.9	-56.2
**OG1RF_11464**	hypothetical protein	-215.7	-64.2
**OG1RF_11252**	hypothetical protein	-590.3	-195.0

Supplementary Table S5. Genes up- or downregulated only in the untreated mutant EFC3C compared to untreated *E. faecalis* wild type.

Locus	Description	Fold change in EFC3C
**Membrane transport**		
OG1RF_12353	iron ABC transporter permease	6.4
OG1RF_10136	ferrichrome ABC transporter substrate-binding protein	4.2
**Carbohydrate metabolism**		
OG1RF_11753	PTS maltose transporter subunit IIBC	-4.3

Supplementary Table S6. Genes up- or downregulated only in the untreated mutant EFC3Py compared to the untreated *E. faecalis* wild type.

Locus	Description	Fold Change in EFC3Py
**OG1RF_12481**	choline-binding protein	11.1
**OG1RF_10490**	cell wall surface anchor protein	6.6
**OG1RF_10485**	cell wall surface anchor protein	5.0
**OG1RF_10486**	cell surface protein	4.6
**OG1RF_10407**	CRISPR-associated protein Csn2	4.3
**OG1RF_12572**	citrate transporter	4.2
**OG1RF_10487**	cell surface protein	4.1
**OG1RF_10655**	glucose transporter GlcU	-4.1
**OG1RF_10836**	nitrate ABC transporter permease	-4.1
**OG1RF_10835**	taurine ABC transporter ATP-binding protein	-4.6
**OG1RF_11036**	ATPase P	-4.8
**OG1RF_11077**	co-chaperone GrpE	-4.8
**OG1RF_11383**	NADPH:quinone reductase	-4.8
**OG1RF_10516**	acyltransferase	-5.3
**OG1RF_10761**	alpha/beta hydrolase	-5.3
**OG1RF_11076**	HrcA family transcriptional regulator	-5.3
**OG1RF_10996**	Cro/Cl family transcriptional regulator	-5.5
**OG1RF_12210**	potassium transporter Trk	-6.1
**OG1RF_12536**	two-component system sensor histidine kinase	-6.9
**OG1RF_12535**	two-component system response regulator	-7.5
**OG1RF_11452**	ABC transporter substrate-binding protein	-7.9
**OG1RF_12071**	transcriptional regulator	-8.3
**OG1RF_12213**	membrane protein	-9.0
**OG1RF_10653**	two-component system response regulator	-9.3
**OG1RF_11908**	virion core protein	-9.5
**OG1RF_11793**	chaperone protein ClpB	-10.4
**OG1RF_11070**	cell division protein FtsW	-11.8
**OG1RF_11002**	phosphoesterase	-12.6
**OG1RF_11071**	cell division protein FtsW	-17.8
	**Carbohydrate metabolism**	
**OG1RF_10297**	mannitol-specific phosphotransferase enzyme IIA component	-4.6
**OG1RF_10432**	PTS fructose transporter subunit IIC	-4.6
**OG1RF_10434**	tagatose 1,6-diphosphate aldolase	-5.5

**OG1RF_10298**	mannitol-1-phosphate 5-dehydrogenase	-5.6
**OG1RF_10682**	beta-phosphoglucomutase	-6.1
	**Cellular communication**	
**OG1RF_10993**	spermidine/putrescine ABC transporter substrate-binding protein	5.6
**OG1RF_12366**	peptide ABC transporter substrate-binding protein	5.5
**OG1RF_10991**	spermidine/putrescine ABC transporter permease	5.1
	**Folding, sorting and degradation**	
**OG1RF_10584**	signal peptidase I	6.5
**OG1RF_11078**	molecular chaperone DnaK	-4.7
	**Glycan biosynthesis and metabolism**	
**OG1RF_12158**	cell division protein FtsI	-4.1
	**Membrane transport**	
**OG1RF_10897**	glutamine ABC transporter substrate-binding protein	11.5
**OG1RF_10895**	amino acid ABC transporter permease	9.6
**OG1RF_10896**	glutamine ABC transporter permease	9.0
**OG1RF_10898**	ABC transporter ATP-binding protein	8.4
	**Nucleotide metabolism**	
**OG1RF_10994**	adenine deaminase	4.3
	**Signal transduction**	
**OG1RF_10760**	membrane protein	-6.2
	**Translation**	
**OG1RF_10025**	RpiR family transcriptional regulator	5.1
	**Hypothetical protein**	
**OG1RF_10796**	hypothetical protein	8.7
**OG1RF_12152**	hypothetical protein	7.9
	hypothetical protein	5.8
**OG1RF_10491**	hypothetical protein	5.8
**OG1RF_10860**	hypothetical protein	4.4
**OG1RF_10861**	hypothetical protein	4.1
**OG1RF_10482**	hypothetical protein	-4.6
**OG1RF_12166**	hypothetical protein	-5.1
**OG1RF_10445**	hypothetical protein	-5.2
**OG1RF_11254**	hypothetical protein	-5.5
**OG1RF_10446**	hypothetical protein	-7.4
**OG1RF_11910**	hypothetical protein	-8.5
**OG1RF_11909**	hypothetical protein	-9.6
**OG1RF_12471**	hypothetical protein	-10.4

**Locus**	**Description**	**Fold change in EFC3Py**
**OG1RF_10838**	manganese transporter	-5.9
**OG1RF_10605**	aldo/keto reductase	-4.3
**OG1RF_11643**	universal stress protein UspA	-4.1
**OG1RF_12470**	cobalamin synthesis protein CobW	4.1
**OG1RF_10878**	adhesin	4.4
**OG1RF_11522**	multidrug MFS transporter	4.6
**OG1RF_10589**	cation transporter	5.6
**OG1RF_10328**	peptidase M23	6.3
**OG1RF_11002**	phosphoesterase	6.6
**OG1RF_11377**	conjugal transfer protein TraX	6.7
**OG1RF_10550**	polysaccharide lyase	9.5
**OG1RF_11767**	multidrug ABC transporter ATP-binding protein	699.5
**OG1RF_11766**	multidrug ABC transporter ATP-binding protein	787.2
**Cellular communication**		
**OG1RF_12368**	peptide ABC transporter permease	-5.2
**OG1RF_12367**	peptide ABC transporter permease	-4.8
**OG1RF_12369**	peptide ABC transporter ATP-binding protein	-4.6
**OG1RF_12366**	peptide ABC transporter substrate-binding protein	-4.1
	**Membrane transport**	
**OG1RF_11677**	ABC transporter ATP-binding protein	-8.6
**OG1RF_11678**	membrane protein	-7.3
**OG1RF_10684**	PTS glucose transporter subunit IIABC	-7.1
**OG1RF_10297**	mannitol-specific phosphotransferase enzyme IIA component	-6.1
**OG1RF_11679**	manganese ABC transporter substrate-binding protein	-5.6
**OG1RF_12479**	PTS mannose transporter subunit IID	-4.7
**OG1RF_12472**	zinc-binding protein	4.8
**OG1RF_12353**	iron ABC transporter permease	5.9
**OG1RF_12354**	iron ABC transporter permease	6.0
**OG1RF_12351**	iron ABC transporter substrate-binding protein	8.0
**OG1RF_10137**	ferrichrome ABC transporter ATP-binding protein	8.1
**OG1RF_12352**	iron ABC transporter ATP-binding protein	8.4
**OG1RF_10136**	ferrichrome ABC transporter substrate-binding protein	13.4
	**Translation**	
**OG1RF_10021**	PTS mannose transporter subunit IID	-4.4
**OG1RF_10020**	PTS alpha-glucoside transporter subunit IIBC	-4.3
**OG1RF_12469**	50S ribosomal protein L33	4.3
	Hypothetical protein	
**OG1RF_10881**	hypothetical protein	20.5

Supplementary Table S7. Differential expression of genes EFC3C treated with COE1-3Py in comparison to the untreated strain.

Locus	Description	Fold change in EFC3C
**OG1RF_12420**	large-conductance mechanosensitive channel	-3.6
**Cellular communication**		
**OG1RF_10993**	spermidine/putrescine ABC transporter substrate-binding protein	4.8
**OG1RF_10434**	tagatose 1,6-diphosphate aldolase	5.1
**Membrane transport**		
**OG1RF_11677**	ABC transporter ATP-binding protein	-7.8
**OG1RF_10432**	PTS fructose transporter subunit IIC	5.5

Supplementary Table S8. Differential expression of genes EFC3Py treated with COE1-3C in comparison to the untreated EFC3Py**.**

Locus	Description	Fold change in EFC3Py
**OG1RF_11766**	multidrug ABC transporter ATP-binding protein	28.3
**OG1RF_11767**	multidrug ABC transporter ATP-binding protein	27.4

Supplementary Table S9. List of primers used to construct the plasmids and verify knockout strains.

Primers	Sequence	Description
***liaF*_INF_F**	gatatctgcagaattcccgaagccgatc catttt	Primer used during PCR amplification of *liaF* from EFC3C.
***liaF*_INF_R**	taccgagctcggatccatatggttcagg agtttctgt	Primer used during PCR amplification of *liaF* from EFC3C.
***liaR*_INF_F**	gatatctgcagaattcgagaacgtgttgt cggc	Primer used during PCR amplification of *liaR* from EFC3Py.
***liaR*_INF_R**	tgattacgccaagctctgccgccaaaac gtcct	Primer used during PCR amplification of *liaR* from EFC3Py.
***liaFcomp*_INF_F**	gcttgatatcgaattttaggcgaccaagt gattat	Primer used during PCR amplification of *liaF* from OG1RF.
***liaFcomp*_INF_R**	tagaactagtggatctcaagcataatctg gaacatcatatggataagcgtaatctgg aacatcgtatgggtatacacggatcacc tcaac	Primer used during PCR amplification of *liaF* from OG1RF.
***liaRcomp*_INF_F**	gtgaggaactgaattcgtgatcaaagta atgttagtggatgacc	Primer used during PCR amplification of *liaR* from OG1RF.& to check gene insertion
***liaRcomp*_INF_R**	acctgttgaatgatctgcagttaagcata atctggaacatcatatggataagcgtaat ctggaacatcgtatgggtatttggctaaa ccatg	Primer used during PCR amplification of *liaR* from OG1RF and to check gene insertion.
**M13F(-20)**	gtaaaacgacggccagt	Forward primer to check insertion in pGCP123
**M13F(-40)**	gttttcccagtcacgac	Forward primer to check insertion in pGCP213
**M13R-pUC (-26)**	caggaaacagctatgac	Reverse primer to check insertion in pGCP213 & pGCP123

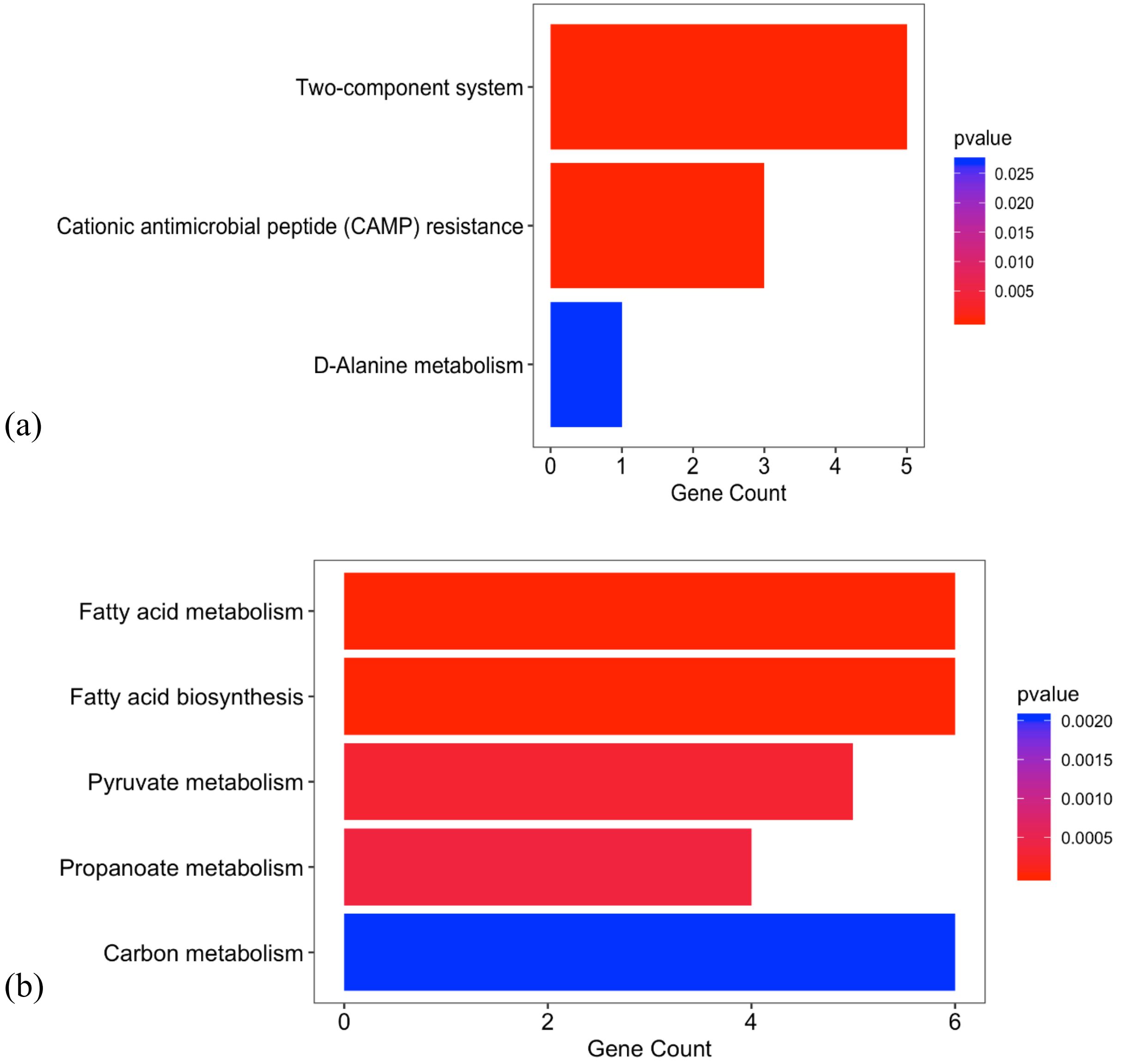

Supplementary Figure S1. Bar plot depicting the most enriched KEGG pathways on treatment of wild type OG1RF with (a) COE1-3C and (b) COE1-3Py. The colours of the bar chart represent the p value of the enriched KEGG pathway. Height represent the gene count.

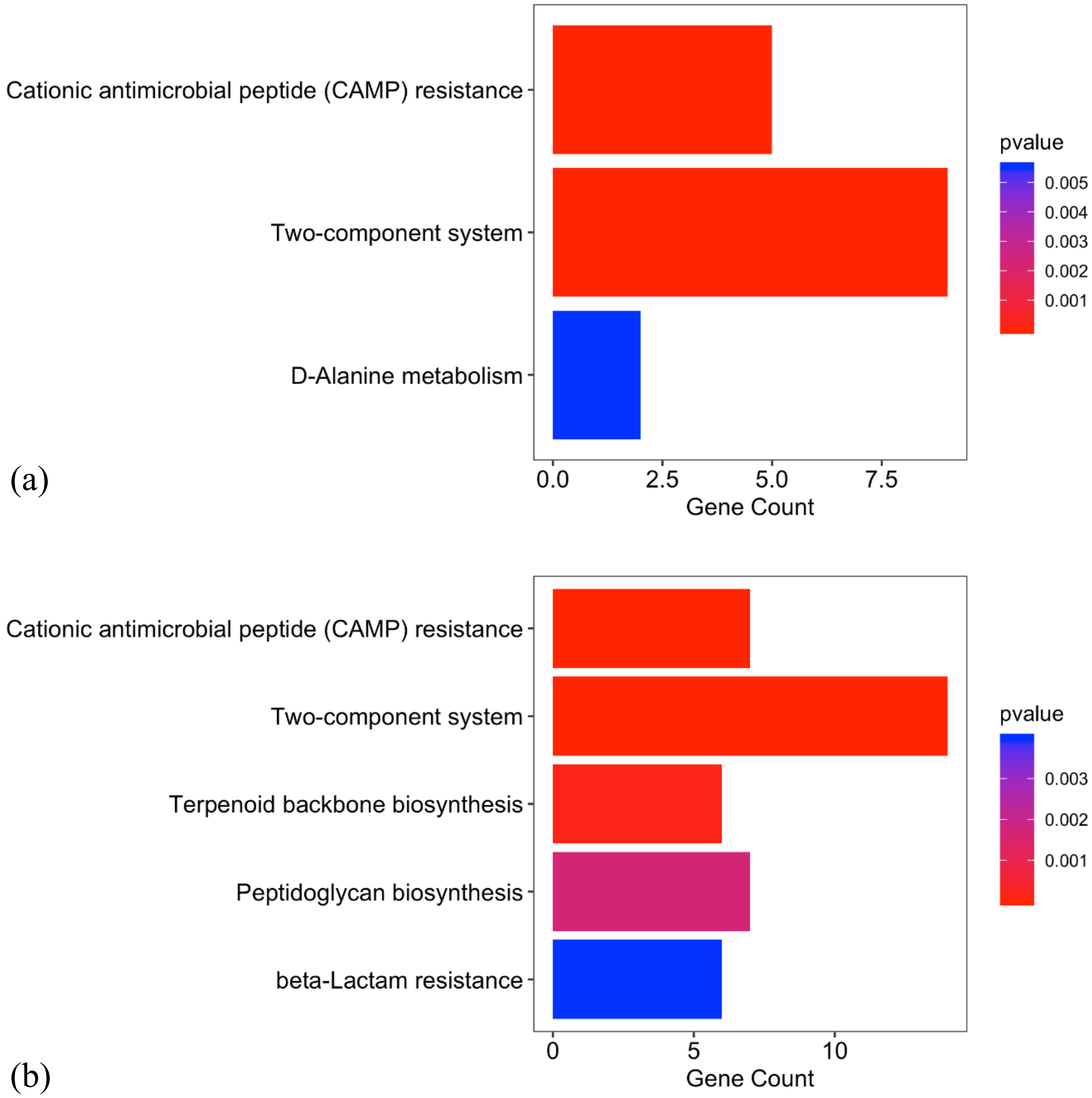

Supplementary Figure S2. Bar plot depicting the most enriched KEGG pathways on treatment of wild type OG1RF with (a) EFC3C and (b) EFC3Py. The colours of the bar chart represent the p value of the enriched KEGG pathway. Height represent the gene count.

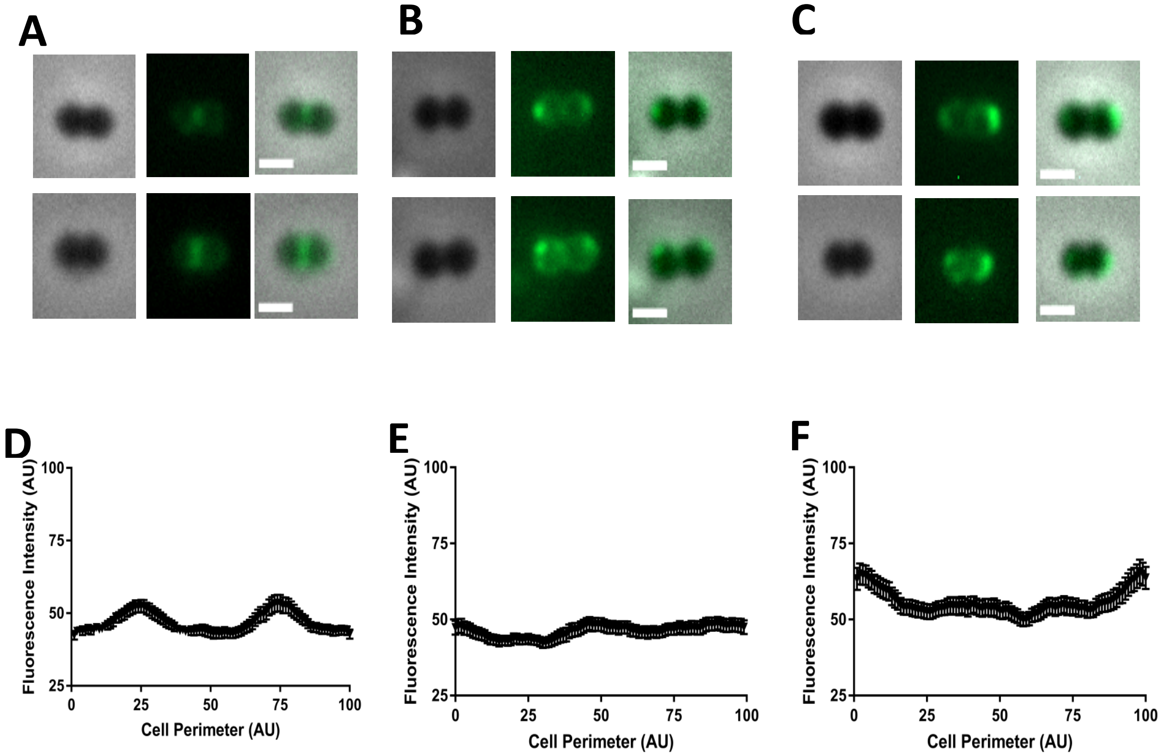

Supplementary Figure S3. Representative images of localization patterns of COE1-3Py in (A&D) *Enterococcus faecalis* OG1RF wild type, (B&E) EFC3C and (C&F) EFC3Py strains. Fluorescence intensity along the circumference of the cell was plotted against the cell perimeter of 1 to 100 units to generate a fluorescence distribution profile. Intensity analysis performed by PSICIC analysis software for two independent experiments with duplicates in each experiment. Excitation and emission wavelengths were 515/640 nm, respectively. The scale bar represents 0.5 um.

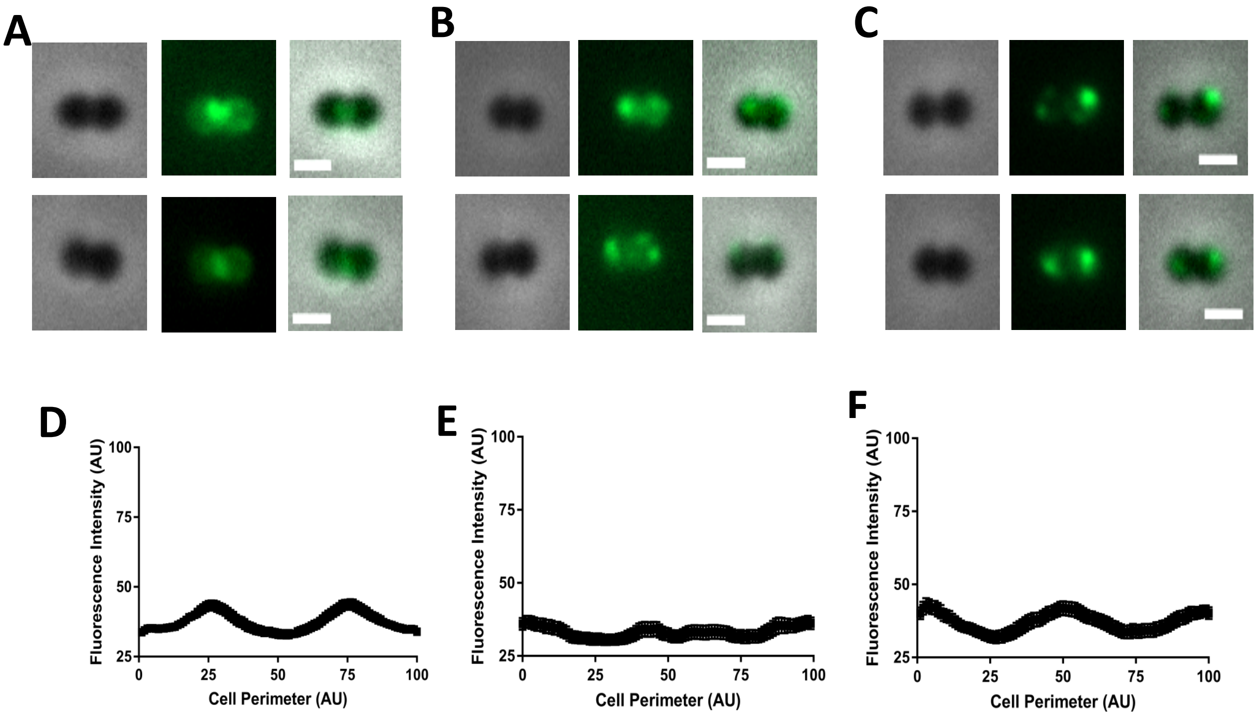

Supplementary Figure S4. Representative images of localization patterns of COE1-3C in (A&D) *Enterococcus faecalis* OG1RF wild type, (B&E) EFC3C and (C&F) EFC3Py strains. Fluorescence intensity along the circumference of the cell was plotted against the cell perimeter of 1 to 100 units to generate a fluorescence distribution profile. Intensity analysis performed by PSICIC analysis software for two independent experiments with duplicates in each experiment. Excitation and emission wavelengths were 515/640 nm, respectively. The scale bar represents 0.5 um.

(A)

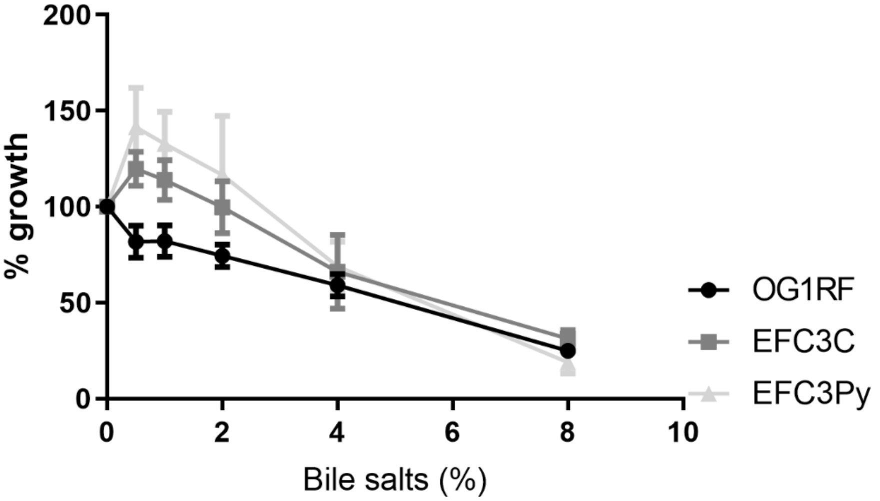

(B)

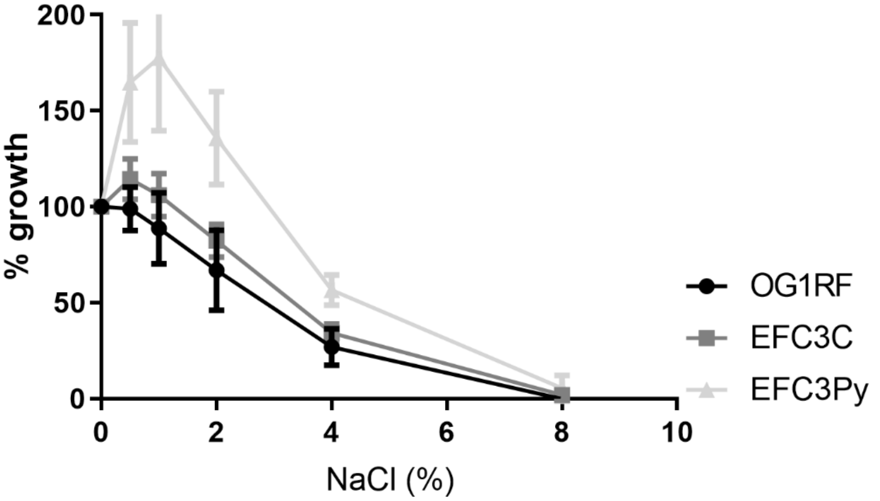

Supplementary Figure S5. Characterization of sodium chloride and bile salts tolerance in EFC3C, EFC3PY and OG1RF wild type. The effect of different concentrations of bile salts (0, 0.5%, 1%, 2%, 4% and 8%) or sodium chloride (0, 0.5%, 1%, 2%, 4% and 8%) was plotted as the % growth against the concentrations of bile salts or sodium chloride (NaCl). This error bars indicate the standard deviations of three biological replicates and three technical replicates (n =9). % growth = (OD_{600} measured at 7 h/ OD_{600} measured at 0 h) x 100. The error bars represent the standard deviation.
